# Modelling nutrient fluxes into the Mediterranean Sea

**DOI:** 10.1016/j.ejrh.2019.01.004

**Published:** 2019-04

**Authors:** Anna Malagó, Fayçal Bouraoui, Bruna Grizzetti, Ad De Roo

**Affiliations:** European Commission, Joint Research Centre (JRC), Ispra, Italy

**Keywords:** Nutrients, Modelling, Mediterranean Sea, Diffuse and point sources, Source apportionment

## Abstract

**Study region:**

Mediterranean River Basins.

**Study focus:**

Human activities and consequent pollution have put the freshwater and marine ecosystems of the Mediterranean region under pressure, with high risk of eutrophication phenomena. In this study, an extended version of the Geospatial Regression Equation for European Nutrient losses model (GREEN), originally developed for estimating nutrient loads from diffuse and point sources in Europe, was extended to include additional nutrient sources using a grid cell discretization. The spatial resolution is 5 arc minute and the model inputs consist of the latest and best available global data.

**New hydrological insights for the region:**

The results of this study show that during 2003–2007 (baseline), 1.87 Tg/y of total nitrogen (TN), 1.22 Tg/y of nitrates (N-NO_3_), 0.11 Tg/y of total phosphorus (TP) and 0.03 Tg/y of orthophosphate (P-PO_4_) were discharged in the Mediterranean Sea. The source apportionment analysis showed that the main contributor to total nitrogen and nitrate loads is agriculture followed by natural background, while for orthophosphate dominant sources include wastewater and scattered dwellings. Two scenarios were investigated to assess sustainable water and nutrient management options, showing that the reduction of 50% of nitrogen surplus leads to a significant reduction of nitrogen emission in regions characterized by high intensity agriculture, while the upgrading of wastewater treatment plants to tertiary level was more efficient for TP reduction.

## 1 Introduction

The Mediterranean Sea is the largest and deepest enclosed Earth Sea and it is bounded on the north by Europe, on the south by Africa, and on the east by Asia. It communicates with the Atlantic Ocean through the Strait of Gibraltar and with the Black Sea through the Dardanelles.

The Mediterranean is an oligotrophic well oxygenated sea with naturally low nutrient concentration due to the anti-estuarine circulation through the Gibraltar Strait driven by the excess of net evaporation over precipitation (Tanhua et al., 2013). However human activities and consequent pollution have put the freshwater and marine ecosystems of the Mediterranean region under pressure (Grizzetti et al., 2017, Liquete et al., 2016).

Here, the eutrophication phenomena due to nutrient inputs from land-based pollution sources is a major environmental problem and the geographical distribution of eutrophication in the Mediterranean Sea occurs in densely populated areas characterized by intensive economic activities. Severe eutrophication has been reported for several coastal areas including the Gulf of Lions, the Adriatic, Northern Aegean and the South Levantine seas. Karydis and Kitsiou (2012) provided an exhaustive overview of the coastal eutrophication problem in the Mediterranean focusing on the connection with public policies of the Mediterranean States and discussing the national and international legislation. Analysing chlorophyll distribution through remote sensing imagery, the authors showed that the Eastern Mediterranean Sea was ultraoligotrophic with a nitrate to phosphate ratio greater than 20, identifying this sea as the largest phosphorus limited body of water in the global ocean.

Concrete measures for the environmental protection of the Mediterranean Sea were taken in 1975 with the Barcelona Convention and over the last 30 years large scientific efforts focused on characterizing water and contaminant (including nutrients) discharge in the Mediterranean Sea. Detailed nutrient discharges into the Mediterranean were provided by Ludwig et al. (2009), who reconstructed the spatial and temporal variability of sources of freshwater and nutrients in the Mediterranean Sea based on a review of available data. Ludwig et al. (2010) estimated the water and nutrient fluxes in the period 1970–2000 using the IMAGE model (Bouwman et al., 2006) and produced future scenarios at spatial scale of 0.5 × 0.5 degree. Bouraoui et al. (2010) provided an estimation of water runoff to the Mediterranean and its variation from 1980 to 2000 developing a consistent geodatabase suitable for hydrological and water quality modelling using river basins of around 200 km^2^. Grizzetti et al. (2012) assessed the nitrogen pressure on surface waters by point and diffuse sources at European scale in the period 1995–2005, providing annual nutrient fluxes of the main European basins that discharge in the Mediterranean. In addition, the Global NEWS model (Seitzinger et al., 2010) was tested in the Mediterranean region using a grid cell of 0.5 degrees (UNESCO-IOC/UNEP, 2003) providing annual loads of inorganic nitrogen and total phosphorus to the Sea. All these studies provide useful information on water and nutrient discharge in the Mediterranean Sea; yet, they usually consider only total nitrogen and total phosphorus, the key geographic scale is a coarse grid resolution or large river basins, and they refer to year 2000 as baseline. In addition, they are still insufficient in providing a more integrated assessment including food, water and environmental synergies and trade-offs in a ‘nexus’ oriented approach (Biggs et al., 2015).

In this context, our research presents a robust modelling framework for assessing water nutrient pollution in the Mediterranean and evaluating the impact of management strategies on water quality. In particular, our study intends to quantify the more recent nitrogen (total nitrogen and nitrate) and phosphorus (total phosphorus and orthophosphate) fluxes to determine the relative importance of different nitrogen and phosphorus sources and to identify hotspots areas of higher pollution where priority actions should focus. The main innovative aspect of the work resides in the development of a simple conceptual model, GREEN-Rgrid (Geospatial Regression Equation for European Nutrient losses extended to global scale in R software environment; R Core Team, 2011) that uses global readily available data and that explicitly considers the linkages between crop, water, and nutrient management impacts on water quality. The model offers a robust framework for overcoming the limited availability of water quality monitoring data in the southern part of the Mediterranean Sea, and for evaluating alternative scenarios of nutrient reduction.

## 2 Material and method

### 2.1 The Mediterranean area

**Fig. 1:**
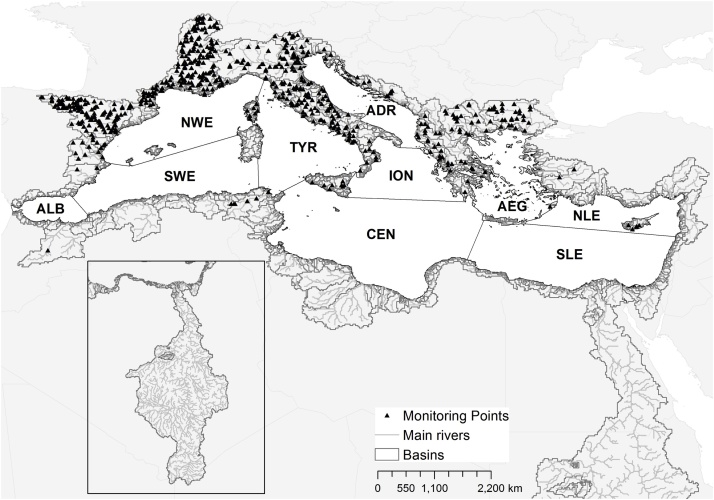
Map of the hydrological network of the major river basins of the Mediterranean area along with the location of the monitoring points. The short names in the Mediterranean Sea refer to ALB: Alboran; SWE: South-Western; NWE: North-Western; TYR: Tyrrhenian; ADR: Adriatic; ION: Ionian; CEN: Central; AEG: Aegean; NLE: North Levantine; SLE: South Levantine.

The Mediterranean Sea covers about 2.5 million km^2^, with an average water depth of 1.5 km. It is commonly divided in 10 sub-basins (Ludwig et al., 2009, Bouraoui et al., 2010, Karydis and Kitsiou, 2012) as shown in Fig. 1: the Western Mediterranean Sea that comprises the Alboran Sea (ALB), the North-Western basin (NWE), the South-Western basin (SWE) and the Tyrrhenian Sea (TYR); the Eastern Mediterranean Sea that covers the Adriatic Sea (ADR) and the Ionian Sea (ION); the Central Mediterranean Sea (CEN); and the Aegean-Levantine Sea that covers the Aegean Sea (AEG), the North Levantine (NLE) Sea and the South Levantine (SLE).

The drainage area of the Mediterranean Sea covers more than 5 million of km^2^, of which 1 million in Europe, 3.8 million in Africa and the rest in Asia. The largest river basins of the Mediterranean Sea are the Nile (Egypt), Rhone (France), Po (Italy), Drin-Buna (Albania), Neretva (Bosnia and Herzegovina), Ebro (Spain), Tiber (Italy), Adige (Italy), Seyhan and Ceyhan (Turkey). Together they account for about half of the average freshwater input by rivers into the Mediterranean Sea (total water discharge of year 2005 was about 9.8 · 10^6^ m^3^/s; Ludwig et al., 2009).

Climatically, the Mediterranean is characterized by warm temperatures, winter-dominated rainfall, dry summers, and a profusion of microclimates due to local environmental conditions (Ludwig et al., 2003). Mean annual temperature follows a marked north-to-south gradient to which local orographic effects are superimposed. The lowest average annual temperatures are found in the higher parts of the Alps (less than 5 °C), while average temperatures above 20 °C are typical for Libya and Egypt. Mean annual precipitation has a general decreasing north-to-south gradient with orography acting as a naturally modulating factor. Annual precipitation values of 1500–2000 mm·yr^−1^ and more are reported in the Alpine and Pyrenean headwater regions of the Po, the Rhone and the Ebro Rivers (Ludwig et al., 2009).

The Mediterranean region is covered by 60% of natural areas (22% forest; 10% shrub and 27% bare), 13% cropland, 8% fodder, 18% grassland and 3% urban areas and water. The main crops are wheat, sorghum, oil crops, maize, barley and temperate fruits. About 17% of the arable land is irrigated (107,000 km^2^) (You et al., 2014) and the average volume of irrigation was 86 · 10^9^ m^3^ considering the available years or the nearest to 2003–2007 (FAO, 2016). The most irrigated crops include wheat, maize, and vegetables.

### 2.2 The GREEN-Rgrid model

The modelling approach used in this study, GREEN-Rgrid, is a conceptual statistical regression model that links nutrient inputs to water quality measurements. It is an extended version of the Geospatial Equation for European Nutrient losses, GREEN, developed by Grizzetti et al., 2005, Grizzetti et al., 2012. The original model was inspired by the spatially referenced regressions on watersheds attributes (SPARROW) methodology (Smith et al., 1997) where the river nutrient load is modelled by non-liner equations linking nutrient sources and basins attributes (Grizzetti and Bouraoui, 2006, Bouraoui and Grizzetti, 2014).

GREEN-Rgrid runs on an annual basis on a grid cell size of 5 min (0.083333 degree, about 10 km at the equator) and can be used to estimate total nitrogen (TN) and phosphorus (TP), nitrate (N-NO_3_) and orthophosphate (P-PO_4_). The model was written in R (R Core Team, 2011) in order to provide a more flexible instrument increasing the reproducibility of the modelling approach and because R is open access widely used programming language.

**Fig. 2:**
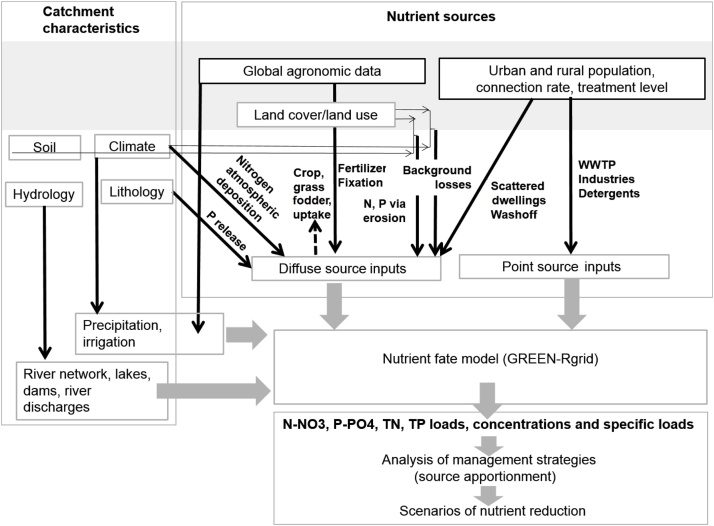
Conceptual framework of the GREEN-Rgrid model.

GREEN-Rgrid consists in a simplified conceptual model, which distinguishes between two different pathways in nutrient transfer from sources to river outlet: nutrients passing through land before reaching the river (diffuse source), and nutrients delivered directly to water bodies (point sources) (Fig. 2). Diffuse sources include the contributions from fertilization (mineral and manure), N fixation, atmospheric deposition (nitrogen), background losses, and scattered dwelling (population not connected to a sewage collecting system). Point sources include the contributions from urban wastewater treatment plants, industries and paved areas.

Diffuse sources transit first through the soil unsaturated and saturated zones before reaching a stream and consequently undergo a preliminary reduction in the soil profile due mostly to the denitrification (nitrogen) and storage processes. Once into the streams or water bodies these nutrients are subject to a second reduction due to algae growth, atmospheric losses, and storage/deposition. Point sources of nitrogen and phosphorus are only retained in streams and lakes. The GREEN-Rgrid considers a routing grid cell structure to establish the emitting-receiving grid cell relationship, where the upstream nutrient load is added as an additional point source to the receiving downstream grid cell.

The load at the outlet of a grid cell is expressed as:

(1) $$L_{i}=[{\text{SUR}}_{i}\cdot S_{i}\cdot R_{i}+({\text{PS}}_{i}+{\text{UL}}_{i})\cdot R_{i}]\cdot (1-{\text{RES}}_{i})$$

where *L* is the annual nutrient load (ton/y), SUR is the nutrient (nitrogen and phosphorus) surplus in the grid cell (ton/y), PS are the point sources (ton/y), UL is the upstream load (ton/y), S, R are the soil and river reduction factors in each grid cell (dimensionless), RES is the nutrient retention in lakes/reservoirs (dimensionless), and *i* represents the grid cell.

Grizzetti et al. (2005), investigating the relationship between nitrogen export and different basin characteristics (annual precipitation, wet season rainfall, temperature, slope, topography, river length, drainage density, annual flow and low flow) based on a Pearson correlation and principal component analysis, identified precipitation as the major descriptor of nitrogen export from the catchment. Based on this analysis, we assumed that the soil reduction factor *S* is a function of precipitation plus irrigation (when present).

The parameter *S* is parametrized as exponential decreasing function as follows:

(2) $$S_{i}=\text{exp}(-\alpha \cdot x_{i})$$

where *x* (dimensionless) is the inverse of the sum of precipitation and irrigation (for each grid cell *i*) normalized by the maximum inverse of the sum of precipitation and irrigation (Frank and Todeschini, 1994) over the entire Mediterranean area, and *α* (dimensionless) is a calibration parameter. The maximum scaling method was used to avoid negative values of the calibration parameter *α* (Grizzetti et al., 2005, Grizzetti et al., 2012, Smith et al., 1997).

The river retention factor *R* is a function of the average area specific runoff (qs, l/s km^2^) and of two calibration parameters *A* and *B* (both dimensionless) that describe the losses in the river network:

(3) $$R_{i}=\frac{1}{1+A\cdot \text{q}{\text{s}}_{i}^{B}}$$

Finally the retention in lakes and reservoirs RES is expressed as:

(4) $${\text{RES}}_{i}=\frac{1}{1+n\cdot \text{H}{\text{L}}_{i}^{C}}$$

where HL is the hydraulic load (m) and *C* (dimensionless) is a calibration parameter, and *n* is a fixed coefficient retrieved from Behrendt et al. (2003).

The specific runoff of rivers and the hydraulic load of lakes were calculated using the annual discharge retrieved from the daily time series (m^3^/s) simulated for the period 2000-2014 by the LISFLOOD model (Van Der Knijff et al., 2010). In particular, the specific runoff was calculated as the ratio between the annual discharge and the upstream area of each grid cell, while the hydraulic load was calculated as the ratio of the streamflow outputs of a lake and its area.

### 2.3 The inputs of the model

The application of the GREEN-Rgrid model requires four basic sets of information for the model parametrization and calibration:

• River basin spatial discretization and water routing structure including lakes location and characteristics;
• Spatial agronomic, hydrologic and climatic characteristics;
• Spatial information on diffuse and point sources of nitrogen and phosphorus;
• Measurements of nutrient loads in surface water.

These inputs were developed from data readily available at global scale, extracting the Mediterranean region for the current application.

#### 2.3.1 Spatial discretization and water routing structure

A global river network and routing structure with 5 arc minute resolution was developed following a procedure described by Olivera et al. (2002). The procedure consists in extracting low resolution (i.e. 5 min) river networks using a high resolution flow accumulation (i.e. 30 s). For applying this procedure, the near-global HydroSHEDS datasets at 30 s of resolution (Hydrological data and maps based on SHuttle Elevation Derivatives at multiple Scales; Lehner et al., 2008) was used. The HydroSHEDS is based on the NASA's Shuttle Radar Topography Mission (SRTM) and provides at various spatial resolution (3, 15 and 30 arc seconds) layers of void-filled DEM, flow direction, flow accumulation, river network and drainage river basins covering up to 60 degrees north and south of the globe. The flow direction with 5 arc minute resolution was subsequently defined based on the 30 s flow direction associated to the 30 s flow accumulation. In areas not covered by HydroSHEDS, we approximated the flow accumulation and flow direction at 5 min with those used by the hydrological LISFLOOD model (Van Der Knijff et al., 2010). Subsequently, the basins and the river network were created in ArcGIS using the upscaled flow direction at 5 min. The river network was then checked comparing the drainage area of the basins of HydroSHEDS and LISFLOOD model at global scale. The global structure that resulted from this process includes 2,158,178 grid cells, and 82,792 river basins with outlet to the sea. For the Mediterranean area, the spatial discretization resulted in 64,148 grid cells and 1770 river basins, of which 550 included more than 5 grid cells (drainage area larger than 270 km^2^). This is in complete agreement with Sadaoui et al. (2018) who, using the same original DEM but a different watershed delineation approach, also discretized the Mediterranean drainage area into 550 watersheds. The remaining basins are single grid cells or small river basins that represent coastal river basins that are very important hotspot areas characterized by high population density.

The global HydroLakes database (Messager et al., 2016) was used in this study for positioning the lakes (natural lakes and reservoirs) on the river network. One single lake was included in each grid cell, and the area of the lakes provided by the HydroLake dataset was considered in the calculation of the lake retention. In the Mediterranean area 671 lakes were included for a total surface area of 97,500 km^2^.

#### 2.3.2 Agronomic, hydrologic and climatic characteristics

The model GREEN-Rgrid uses annual precipitation and irrigation as proxies for the basin retention of nitrogen, and only precipitation for phosphorus. The spatial information on annual precipitation from 2000 to 2014 was obtained from the global historic precipitation dataset MSWEP (Beck et al., 2017). The MSWEP dataset consists of a global high resolution gridded dataset (0.25 degrees) of precipitation obtained by merging satellite information with reanalysis data. The annual irrigation (mm) spatialized at 5 min grid cell level was defined based on a downscaling procedure using the net irrigation volume (m^3^) reported at country level by AQUASTAT (FAO, 2016), the irrigated area in each cell retrieved from SPAM (You et al., 2014), and the difference between the potential evapotranspiration (PET) and precipitation (PCP).

In particular, the annual potential evapotranspiration was estimated as a function of annual temperature according to Langbein (1949):

(5) $${\text{PET}}_{i}=325+(21\cdot T_{i})+(0.9\cdot T_{i}^{2})$$

where PET is the potential evapotranspiration in (mm/y) of year *i*, and *T* is the average annual temperature (°C) of year *i*, retrieved from Dee et al. (2011).

The consumptive water use of irrigation is defined as the volume of water needed to compensate for the deficit between potential evapotranspiration and effective precipitation over the crop growing period. The net irrigation volume at country level was thus spatialized based on the distribution of the difference PET-PCP in irrigated cells.

The SPAM model provides for 42 crops and four levels of agriculture intensification, the crop-specific physical area, harvest area (sum of areas considering multiple-harvest in a year), yield and production (product of yield and harvest area). Data are available for the year 2005 (average of 3 years centered on 2005) for four production systems including irrigated high inputs production, rainfed high inputs production, rainfed low inputs production, rainfed subsistence production (You et al., 2014). The crops used in SPAM are described in Table S1. The sum of the areas of all irrigated crops within each grid cell provided the irrigated area of the cell.

#### 2.3.3 Nutrient inputs from diffuse and point sources

Information on nutrient sources at high spatial resolution is difficult to retrieve or is only partially available at large scale (Grizzetti et al., 2011). For this reason, we developed global spatially explicit layers at 5 min resolution of N and P pressures from anthropogenic and natural sources using the most recent available global spatial datasets for year 2005. These high spatial resolution nutrient inputs are described in detail and in this study we provide a synthesis.

##### 2.3.3.1 Landuse

A landuse dataset was created in order to spatialize the nutrient inputs from agricultural, urbanized and natural areas. The GLOBCOVER 2009 map (Arino et al., 2008) was used to define 10 classes of land cover: cropland, fodder, grassland, forest, shrub, bare, urban area, water, sea, and snow. The extent of the class cropland was constrained using the information of the Spatial Production Allocation Model (SPAM, You et al., 2014) at 5 min resolution. The class fodder was obtained as the areal difference between the aggregated classes of GLOBCOVER representative of cropland and the SPAM cropland. Cropland includes permanent and non-permanent, irrigated and rainfed crops and fodder. Forest, bare and shrub are considered natural areas, while urban areas, water, sea, and snow are considered together as other areas.

##### 2.3.3.2 Diffuse sources

The diffuse sources of N and P considered in this study include nitrogen and phosphorus surplus on cropland and grassland areas, atmospheric deposition in natural areas (natural background), nutrient transported via erosion, nutrient in washoff, phosphorus weathering and scattered dwellings (people not connected to a wastewater collecting system).

The cropland and fodder nitrogen and phosphorus surpluses were calculated as the difference between the sum of mineral and manure fertilizers, plant fixation (only for nitrogen), atmospheric deposition (only for nitrogen) and nutrient uptake by crops and fodder. The nutrient surplus in grassland areas was calculated as the difference between the sum of mineral and manure fertilizers, atmospheric deposition (only for nitrogen) and a 60% of all nutrient inputs (Bouwman et al., 2005).

The N and P mineral fertilizers on cropland at 5 min resolution were obtained by downscaling the total amount of net mineral fertilizers available at country scale from FAOSTAT (FAOSTAT, 2016a) for the year 2005 using the spatial distribution of nutrient crop uptake estimated at grid cell level. The total net amount of N fertilizer by country was calculated as the difference between the gross mineral fertilizers and the total gaseous losses. The gaseous losses of nitrogen were estimated using the emission factor (% NH_3_ losses of N content) reported in Bouwman et al. (1997) and FAOSTAT factors (FAOSTAT, 2016b). A portion of the total fertilization was applied on grassland using the percentages provided by Lassaletta et al. (2014).

We distributed the national net mineral fertilizers in each grid cell on non-leguminous crops based on the nitrogen content of the yield, and on fodder based on the deficit between nitrogen fixation and nitrogen uptake. The remaining fertilizer non applied on crop or fodder was distributed uniformly on grassland areas.

Crop uptake was estimated at grid cell as the product of dry yield (fresh yield reduced using coefficients of crop moisture content from the EPIC model; Williams, 1995) and the nitrogen and phosphorus contents of each crop retrieved from the SWAT model database (Neitsch et al., 2010). The wet yields of FODG were retrieved from FAOSTAT at country level and then converted in dry yields using the moisture content given in Table S1. The nitrogen and phosphorus uptake in grassland was calculated as a 60% of all nutrients inputs (Bouwman et al., 2005).

The nitrogen fixation for each crop was calculated as the product of the specific nitrogen fixation amount in kg N/ha retrieved from literature and the total harvest area of each crop. In addition, we assumed a specific nitrogen fixation of 65 kg/ha and 4 kg/ha for fodder and grassland, respectively.

The amount of nitrogen and phosphorus available in animal manure was computed by multiplying the number of animals by category (retrieved from FAOSTAT, 2016c) by the excretion coefficients by animal category (kg N or P/head/y) obtained from FAOSTAT (FAOSTAT, 2016d). This approach is similar to that proposed by Bouwman et al. (2013) and Liu et al. (2010). We have taken into account the NH_3_, N_2_O and NO gaseous losses during excretion using specific volatilization rates of different livestock categories reported in Bouwman et al. (1997) and FAOSTAT (FAOSTAT, 2016e). The national total amount of stables and meadows manure was distributed on different landcover classes using specific manure application limits.

Nitrogen atmospheric deposition was retrieved from global rasters of 1 degree resolution from the World Data Centre for Precipitation Chemistry web site (http://wdcpc.org/). The distribution of total nitrogen deposition between the landcover classes in each grid cell was proportional to the area of each class.

The phosphorus release from weathering was calculated based on the lithological map GLIM (Hartmann and Moosdorf, 2012), that provides 16 lithological classes for the whole globe, assigning values of specific P release (kg P km^2^/y) for each of the classes as described in Hartmann et al. (2014). The total value of P release at 5 min grid cell was obtained summing the P release obtained for each lithological class present in each grid cell.

Nutrient emissions from built-up areas (washoff) were calculated at grid cell level as the product of urban population and the specific N and P emission for inhabitant proposed by De Wit (2000).

N and P emissions via erosion into surface waters were calculated as the product of three factors: (i) soil erosion loss (ton/y), (ii) nutrient content in the top soil, and (iii) sediment delivery ratio. In particular, soil erosion loss was estimated for the period 2000–2014 using the modified RUSLE model (Renard et al., 1997).

##### 2.3.3.3 Point sources

Nutrient inputs produced by human settlements connected to sewers, wastewater treatment plants, industries and paved areas were considered as point sources (hereafter WWTP) in the model and were estimated taking into account urban and rural population, emission rates per person, the percentage of urban and rural population connected to wastewater treatment plants and their treatment level. The methodology to define these nutrient specific emissions from points sources (kg/person) at grid cell level consisted in determining the nutrient point sources at country level and then to downscale them using population density as spatial proxy. The product of nutrient specific emission, the connected population and the treatment level efficiency resulted in the gridded point sources map at 5 min. We distinguish the rural and urban population inside each grid cell using the GHSL datasets (Dijkstra and Poelmann, 2014) available for year 2015 at resolution of 1 km. The original data was rescaled for the year 2005 using the FAOSTAT population values (FAOSTAT, 2016f).

Specific domestic emissions were estimated according to the methodology described by Grizzetti et al. (2012) considering human protein intake, the percentage of food waste, the connection rates and the urban and rural population. Point source emissions from industries were estimated as 15% of human emissions as suggested by Morée et al. (2013), while phosphorus from detergents was calculated based on a relationship between STP (sodium tri-phosphate)–detergents consumed by selected countries and the GDP (Gross Domestic Product) for year 2005. People not connected to a sewage collecting system were considered as scattered dwellings and their emissions were added to the pool of diffuse nutrient inputs.

A summary of the major inputs used by the model for the major river basins discharging into the Mediterranean Sea is given in Table 1, Table 2 for nitrogen and phosphorus, respectively.

**Table 1:** Nitrogen inputs from diffuse and point sources for large Mediterranean basins along with the long-term average simulated loads for the period 2003–2007.

		Diffuse sources						Point sources			
Basins	Area (1000 km^2^)	Surplus on cropland and fodder (ton/y)	Surplus on grassland (ton/y)	Nutrient via erosion (ton/y)	Scattered dwellings (ton/y)	Natural background^c^ (ton/y)	Nutrient via washoff (ton/y)	WWTP (ton/y)	Industries (ton/y)	TN simulated (ton/y)	N-NO_3_ simulated (ton/y)
Nile	3,182	1,919,647	882,610	861,989	193,834	2,959,809	16,537	46,351	6,325	642,213^b^	585,050^b^
Rhone	97	42,416	76,668	24,427	3,141	52,339	2,773	13,322	1,353	88,531^a^	40,598^a^
Ebro	86	233,681	30,263	14,607	243	51,018	902	5,428	571	46,243	36,916
Po	73	230,091	46,153	27,755	1,186	38,978	5,509	34,941	4,278	166,552^a^	86,295^a^
Evros-Maritsa	53	80,086	9,598	6,676	1,741	22,987	816	5,568	603	37,229	27,164
Medjerda	24	24,598	7,351	2,951	1,446	2,996	446	2,738	314	9,018	4,973
Other basins	1,428	1,843,572	300,361	271,761	65,345	586,304	43,397	288,852	32,751	855,532	399,635
**Total**	**4,943**	**4,374,090**	**1,353,003**	**1,210,166**	**266,936**	**3,714,431**	**70,379**	**397,201**	**46,195**	**1,845,318**	**1,180,631**

^a^Simulated values at the last available monitoring points before the outlets.

^b^These values do not consider water abstraction occurring in the Nile Delta.

^c^Natural background includes the atmospheric deposition in natural areas.

**Table 2:** Phosphorus inputs from diffuse and point sources for large Mediterranean basins and long-term average simulated loads for the period 2003–2007.

		Diffuse sources						Point sources				
Basins	Area (1000 km^2^)	Surplus on cropland and fodder (ton/y)	Surplus on grassland (ton/y)	Nutrient via erosion (ton/y)	Scattered dwellings (ton/y)	Nutrient via washoff (ton/y)	Phosphorus weathering (ton/y)	WWTP (ton/y)	Industries (ton/y)	Detergents (ton/y)	TP simulated (ton/y)	P-PO_4_ simulated (ton/y)
Nile	3,182	44,929	124,848	319,584	36,394	3,758	24,518	7,344	1,003	591	7,437^b^	7,197^b^
Rhone	97	–	8,987	9,812	404	630	883	1,178	119	613	1,938^a^	1,465^a^
Ebro	86	43,656	5,431	6,275	32	205	932	523	55	314	495	347
Po	73	17,855	7,581	8,262	167	1,252	635	3,971	486	429	4,757^a^	2,547^a^
Evros-Maritsa	53	5,544	976	2,961	275	186	332	760	82	217	1,208	643
Medjerda	24	18,071	1,026	1,684	261	101	404	387	45	104	1,044	290
Other basins	1,428	159,549	36,628	120,932	11,020	9,863	18,907	38,845	4,356	10,377	92,100	17,025
**Total**	**4,943**	**289,604**	**185,476**	**469,511**	**48,553**	**15,995**	**46,612**	**53,010**	**6,146**	**12,646**	**108,506**	**29,987**

^a^Simulated values at the last available monitoring points before the outlets.

^b^These values do not consider water abstraction occurring in the Nile Delta.

#### 2.3.4 Monitoring network

Times series of annual loads of nitrate, orthophosphate, total nitrogen and total phosphorus are needed for the calibration of the model GREEN-Rgrid. We collected time series of daily and annual nutrient concentrations from different sources for the period 2000–2014. At global scale we retrieved annual data from the GEMS/Water Data Center (Meybeck and Ragu, 1997); at European scale the data provided by the European Environmental Agency (WATERBASE, 2016) and the JRC internal database were used (Grizzetti et al., 2012). Other water quality data, usually average annual data, were retrieved directly from literature in particular for Northern African countries (Aissaoui, 2013, Guasmi et al., 2012, Merzoug and Merazing, 2012, Taybi et al., 2016, Brahimi et al., 2014, Makhoukh et al., 2011). The final monitoring dataset for the period 2003–2007 consists in about 500 monitoring points (Fig. 1) with 1661, 1077, 1367 and 1085 data entries of annual concentration of N-NO_3_, TN, P-PO_4_ and TP, respectively. For Northern African countries and Turkey, we managed to collect 9 values of annual nitrate concentration and 4 values for orthophosphate.

Albeit the efforts in collecting monitoring data, these monitoring stations resulted limited in space covering mainly the Rhone River Basin with around 28% of all measurements, the Po with 15% of TP and TN observations, and the Ebro with around 28% of N-NO_3_ and P-PO_4_ measurements. The Nile River is the largest ungauged basin in the Mediterranean. Similar problems of limited availability of water quality data were reported by Ludwig et al. (2009) during their assessment of nutrient discharge into the Mediterranean Sea. However, it must be stressed that the data collected in this study is significantly richer than that used by Ludwig et al. (2009) who limited their data collection at the outlets of the major rivers.

The daily time series of TN, TP, N-NO_3_ and P-PO_4_ were considered only if at least 8 values per year were available. In addition, when total nitrogen was not available, it was calculated as the sum of Total Kjeldahl Nitrogen, nitrates and nitrites. Finally, we checked that for any specific monitoring point and for any specific period the values of N-NO_3_ and P-PO_4_ were lower than TN and TP, respectively. The annual concentration was then converted to annual load multiplying the concentration with the flow obtained from LISFLOOD (Van Der Knijff et al., 2010).

### 2.4 Calibration method and extrapolation

The model runs on an annual basis and it was calibrated using a sequential approach. We first calibrated N-NO_3_. Then we calibrated the difference between TN and N-NO_3_ load as estimated in the previous calibration. Similarly, we calibrated P-PO_4_ and then the difference between TP and P-PO_4_ imposing the calibrated P-PO_4_ load. This procedure was followed to ensure consistency between the estimation of the total nutrient loads and their various components.

The calibration of the model was performed for the period 2003–2007 using a Latin Hypercube algorithm. Only the basins with available monitoring data were selected during the calibration. The calibration of the nutrient components was performed finding the optimal values of model parameters using the ideal point error (IPE) approach that is a dimensionless composite index that measures model performance with respect to an ideal point in a *n*-dimensional space where *n* is the number of model performance evaluation metrics employed. An IPE value of zero corresponds to a perfect ideal point. Our IPE index integrates five popular metrics and is defined as follows:

(6) $$IPE={\left[0.2\cdot \left({\left(\frac{RMSE}{\text{max}(RMSE)}\right)}^{2}+{\left(\frac{NSE-1}{\text{min}(NSE-1)}\right)}^{2}+{\left(\frac{bR^{2}-1}{\text{min}(bR^{2}-1)}\right)}^{2}+{\left(\frac{1-(RSR-\text{max}(RSR))}{\text{min}(RSR-\text{max}(RSR))}\right)}^{2}+{\left(\frac{1-(absPBIAS-\text{max}(absPBIAS))}{\min(\text{absPBIAS}-\max(\text{absPBIAS}))}\right)}^{2}\right)\right]}^{0.5}$$

where RMSE is the Root Mean Square Error, the NSE is the Nash Sutcliffe coefficient (Nash and Sutcliffe, 1970), *bR*^2^ is the coefficient of determination (*R*^2^) multiplied by the slope of the regression line between simulated and observed; RSR is the ratio of the RMSE between simulated and observed values and the standard deviation of the observations, and PBIAS is the percent bias between simulated and observed values. These statistics were also used to investigate the robustness of the model calibration in addition to graphical plots of the predicted versus measured loads showing the agreement between measurement and model estimations.

The coefficient of determination (*R*^2^) multiplied by the slope of the regression line between simulated and observed (*bR*^2^) indicates the degree of linear relationship between simulated and observed data. A *bR*^2^ value close to one indicates a better performance. However, it is very sensitive to high values. The Nash–Sutcliffe efficiency (NSE) is a normalized statistic that ranges from −∞ to 1, with 1 indicating a perfect match. NSE tends to give more importance to higher values and lesser importance to lower values (Legates and McCabe, 1999). Percent bias (PBIAS) is an error index that calculates the average tendency of the simulated data to be either larger or smaller than their observed counterparts with zero indicating the optimal value (Gupta et al., 1999). Positive values indicate overestimation bias, whereas negative values indicate model underestimation bias (Zambrano-Bigiarini, 2017). RSR combines the feature of an error index, RMSE, and a normalization factor so that it can be applied to various constituents (Moriasi et al., 2007). RSR ranges from the optimal value of 0 to infinity with smaller values indicating a better fit.

The IPE was adjusted to give a major weight to the three river basins where more data were available (Po, Rhone and Ebro). We defined thus the IPEw as follows:

(7) $$\text{IPEw}={\left({\text{IPE}}_{\text{group}}\cdot {\text{IPE}}_{\text{Po}}\cdot {\text{IPE}}_{\text{Rhone}}\cdot {\text{IPE}}_{\text{Ebro}}\right)}^{1/4}$$

where IPE_group_ was calculated considering all the basins with data excluding the Po, Rhone and Ebro.

The calibration was performed considering all the available data due to the limited number of measurements in North Africa. The procedure was organized according to the following steps:

a) Calibration of N-NO_3_ and TN:
  • Estimate the load of nitrate originating from atmospheric deposition in natural areas in basins with fraction of natural areas (forest + bare + shrub) greater than 50% (this fraction is identified with the parameter *η*); 1000 runs were performed; the optimal value was found calculating the IPE based on the comparison between the annual observed and simulated N-NO_3_ loads;
  • Using the calibrated *η* for N-NO_3_, estimate the optimal *α* (Eq. (2)), *A* (Eq. (3)), *B* (Eq. (3)), and *C* (Eq. (4)), for N-NO_3_ in all basins with available observed loads. 1000 runs were performed; the optimal values were found calculating the IPEw based on the comparison between the annual observed and simulated N-NO_3_ loads;
  • Using the previous calibrated dataset for N-NO_3_ (*η*, *α*, *A*, *B* and *C*), estimate the optimal *η* for TN in the basins with fraction of natural areas (forest + bare + shrub) greater than 50%; 1000 runs were performed; the optimal value was found calculating the IPE based on the comparison between the annual observed and simulated TN loads;
  • Using the calibrated *η* for TN, estimate the optimal, *A*, *B* and *C* for TN minus N-NO_3_ previously calibrated in all basins with data available. 1000 runs were performed; the optimal values were found calculating the IPEw based on the comparison between the annual observed and simulated TN loads;
b) Calibration of P-PO_4_ and TP:
  • Estimate the optimal *α*, *A*, *B* and *C* for P-PO_4_ in all basins with data available. 1000 runs were performed; the optimal values were found calculating the IPEw based on the comparison between the annual observed and simulated P-PO_4_ loads;
  • Using the previous calibrated dataset for P-PO_4_ estimate the optimal *α*, *A*, *B* and *C* for TP minus P-PO_4_ in all basins with data available. 1000 runs were performed; the optimal values were found calculating the IPEw based on the comparison between the annual observed and simulated TP loads.

Finally, the parameter values obtained during the calibration were used to extrapolate the model estimation in all the grid cells of the Mediterranean region. Due to the limited data availability, all monitoring data was used during the calibration process, so the model could not be validated. It must be stressed that the modelled results obtained in the southern basins should be interpreted with care because of the scarcity of monitoring data. These results could be significantly improved with the development of effective monitoring programmes to track improvement of water quality (Metcalfe et al., 2017).

Finally, it must be pointed out that the calibrated parameter set is unique for the whole Mediterranean but the retention factors are specific to a grid cell since we use in Equations 2 to 5 the precipitation and irrigation amount as well as the discharge specific to each cell.

### 2.5 Sensitivity analysis of model parameters

The sensitivity analysis of the model parameters was assessed using dotty plots. These are plots of parameter values of relative changes versus the objective function (IPE_w_). The main purpose of these graphs is to show the distribution of the sampling points as well as to give an idea of the parameter sensitivity. They have the advantage to help identifying the most appropriate range to achieve the highest performance, and thus reducing the uncertainty in model prediction. In addition, the sensitivity of each parameter with respect to the others was assessed, relating the randomly generated parameters (Latin Hypercube) against the objective function value as follows:

(8) $$g=\mu +\sum_{i=1}^{m}\beta_{i}\cdot b_{i}$$

where *g* is the objective function, *b* is the parameter, *μ* and *β* are the coefficients of regression and *m* is the number of simulations. The *t*-value and the significance level of each parameter were used to identify the relative significance of each parameter *b*_*i*_.

### 2.6 Management strategies and scenarios of nutrient reduction

Quantifying the amount of nitrogen and phosphorus discharged to the coastal waters and the identification of their sources in the river basin is crucial to plan management strategies for the protection of coastal ecosystems and their services. The modelling framework presented in this study allows quantifying nutrient fluxes to the sea considering the contribution of natural processes and different sources, such as agriculture domestic and industries. In particular, nitrogen and phosphorus losses in soil, rivers and lakes are estimated capturing all natural processes responsible for nutrient retention in the river basin. In addition, the contribution of different sectoral sources is investigated to identify efficient and targeted nutrient reduction strategies. Two scenarios of nutrient control were investigated: nutrient surplus reduction by 50% (S1); and improvement of WWTP treatment efficiency assuming that all wastewater is treated to the tertiary level (S2). S1 consists in the reduction of the nutrient surplus in each grid cell in the Mediterranean region and thus it is assumed that crop yield is not affected as all crops still receive fertilizers in excess of their requirements. S2 increases where possible the treatment efficiency at the tertiary level, this is expected to have high impact in the southern part of the Mediterranean Sea where a large portion of the collected wastewater is discharged directly into the sea without any treatment (Powley et al., 2016).

## 3 Results

### 3.1 Results of the model calibration

The final results were obtained after the application of the sensitivity analysis. The analysis of the dotty plots for each parameter of the calibrated variables has allowed reducing the ranges of sampling, thus reducing the uncertainty in the estimation of the optimal parameter values.

Table 3 shows the reduced ranges with respect to their initial values and the optimal parameters obtained after calibration. The relative sensitivity analysis of the parameters for each variable (Table 4) showed that the most sensitive parameters for nitrates, total phosphorus are *A* and *B* which are related to river retention, while orthophosphate and total nitrogen are mostly controlled by *α* and *C* which are related to soil and lake retention.

**Table 3:** Values of parameters estimated by the model calibration in the Mediterranean area.

Determinand	α		A		B		C		η	
	Range	Optimal	Range	Optimal	Range	Optimal	Range	Optimal	Range	Optimal
N-NO_3_	0.1–10	5.59	0.1–10	0.88	0.1–5	4.62	0.1–1	0.59	0.1–0.5	0.34
TN	0.1–10	8.39	0.1–10	6.7	0.1–5	2.95	0.1–1	0.96	0.34–0.6	0.39
P-PO_4_	10–20	14.98	0.1–10	8.59	3–5	4.24	0.1–0.5	0.49	NA	
TP	1–7	1.89	0.1–10	0.84	0.1–1	0.76	0.1–0.5	0.11	NA

**Table 4:** Sensitivity analysis of the parameters related to retention. The relative sensitivity rank for each parameter is reported in the column “rank” with values from 1 (“High sensitivity”) to 4 (“Low sensitivity”). Only the parameters with significance level <0.01 are reported.

Determinand	Type	*t*-value	Retention	Rank
N-NO_3_	*B*	−48.055	River retention	1
	*A*	10.893	River retention	2
	*α*	9.339	Soil retention	3
	*C*	−3.786	Lakes retention	4

TN	*α*	−18.707	Soil retention	1
	*C*	4.266	Lakes retention	2
	*B*	2.602	River retention	3

P-PO_4_	*α*	−17.582	Soil retention	1
	*C*	−2.635	Lakes retention	2

TP	*A*	11.633	River retention	1
	*B*	−9.94	River retention	2
	*C*	6.808	Lakes retention	3

**Fig. 3:**
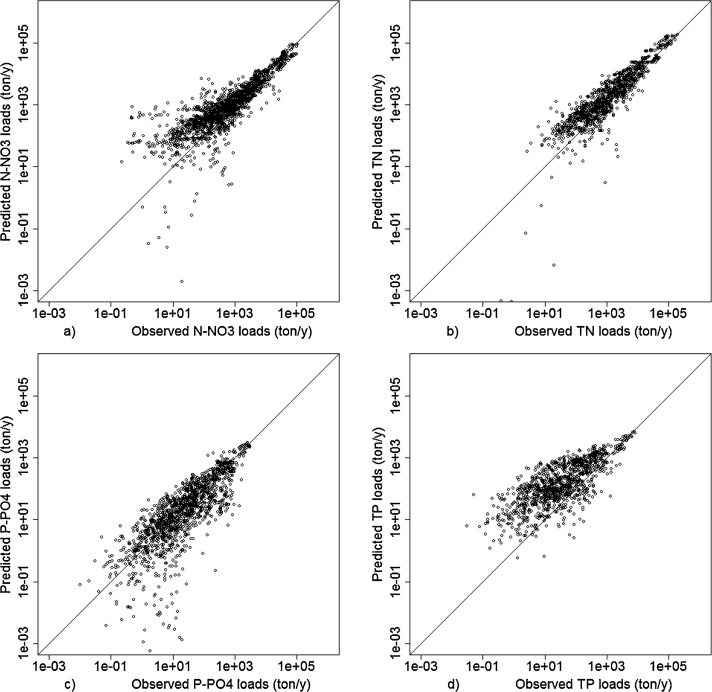
Comparison of modelled and measured loads for N-NO_3_ (a), TN (b), P-PO_4_ (c) and TP (d).

The model calibration yielded results in good agreement with the measured data for each of the calibrated variables (Fig. 3 and Table 5). The calibration of nitrate resulted better than the calibration of phosphorus due to the higher mobility of N in water compared to P and the complex chemical properties of P in natural waters (Jarvie et al., 2002). The Nash–Sutcliffe efficiency is good for all determinands. The analysis of the PBIAS indicates that the model tends to overestimate TN and TP, and slightly underestimate N-NO_3_ and P-PO_4_. This was observed also in Grizzetti et al. (2008). A similar trend is found in the African and Asian basins.

**Table 5:** Model performances for each modelled determinand. The performances were calculated comparing simulated and observed annual loads.

Determinand	Monitoring points (# data entries)	NSE	PBIAS (%)	*bR*^2^	RMSE	RSR	IPEw
N-NO_3_	587 (# 1661)	0.85	−8.6	0.69	4550	0.37	0.16
TN	514 (# 1077)	0.81	28.2	0.78	9009	0.42	0.21
P-PO_4_	564 (# 1367)	0.76	−10.4	0.66	177	0.49	0.41
TP	519 (# 1085)	0.72	67.2	0.73	433	0.53	0.27

### 3.2 The nutrient fluxes in the Mediterranean Sea

**Fig. 4:**
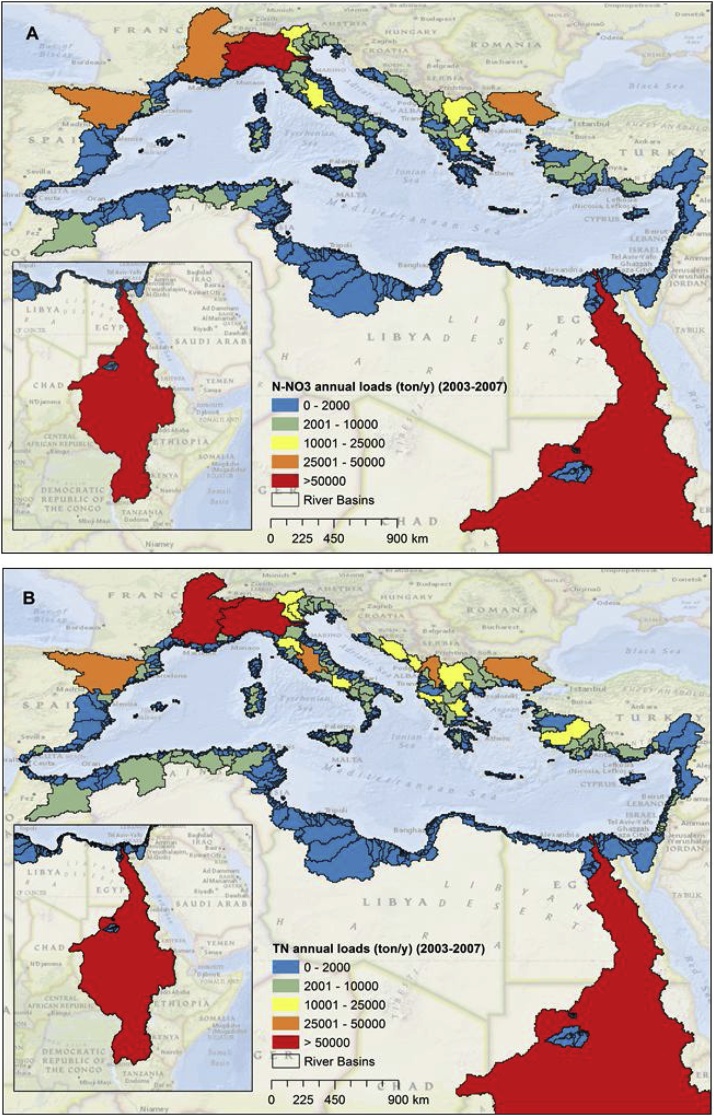
Mean annual river basins discharge of N-NO_3_ (A) and TN (B) for the period 2003–2007.

**Fig. 5:**
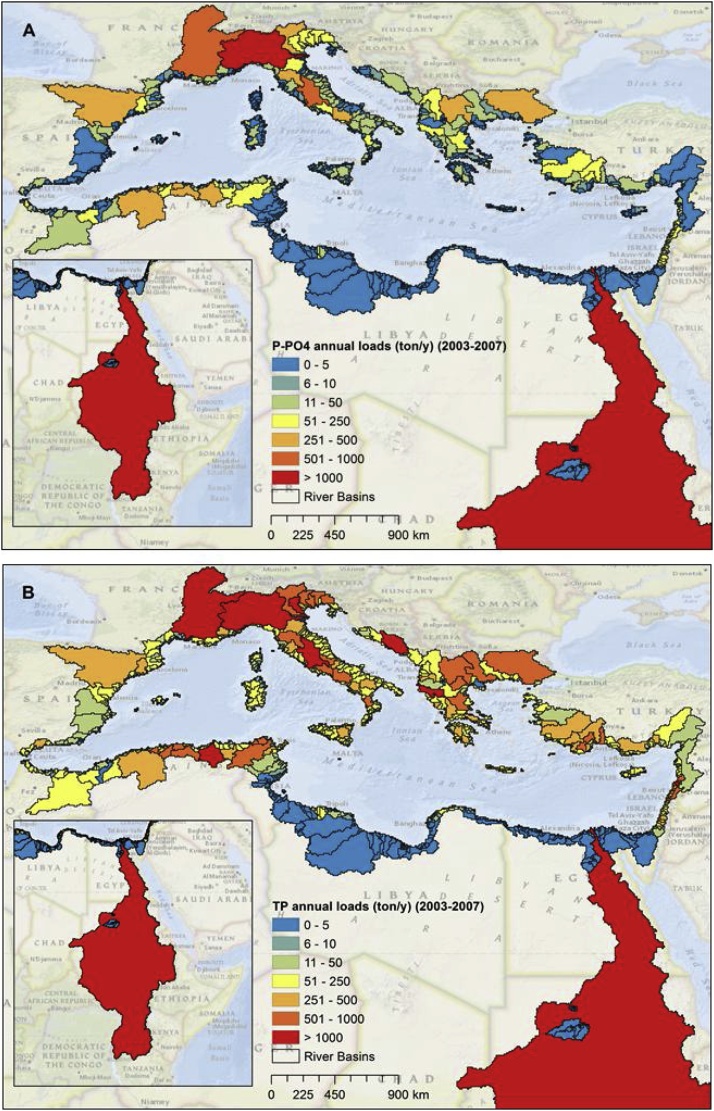
Mean annual nutrient discharge of P-PO_4_ (A) and TP (B) from river basins in the period 2003–2007.

For the period 2003–2007 we estimated that on average, 1.87 Tg/y of TN, 1.22 Tg/y of N-NO_3_, 0.11 Tg/y of TP and 0.03 Tg/y of P-PO_4_ are discharged into the Mediterranean Sea. The Nile, Po, Rhone and Ebro were the main river basins that contribute to the nutrient discharge in the Mediterranean Sea (Fig. 4, Fig. 5, Table 1, Table 2).

**Fig. 6:**
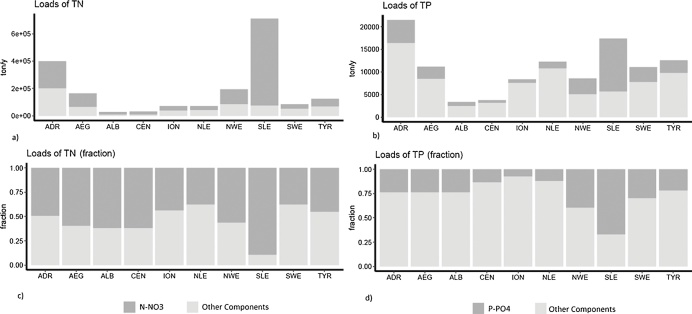
Nutrient loads discharged into ten Mediterranean Sea sub-basins during the period 2003–2007 (total nitrogen (a); total phosphorus (b)). The lower part of the graph shows the fraction of total nitrogen in the nitrate form (c) and the fraction of total phosphorus in the orthophosphate form (d).

We estimated that N-NO_3_/TN and P-PO_4_/TP ratios were respectively around 0.65 and 0.27. These ratios are in good agreement with the studies of Ludwig et al. (2009) and Turner et al. (2003). Hotspots of high biological productivity and eutrophication originating from anthropogenic nutrient inputs are present in coastal areas in the South Levantine (SLE), Adriatic Sea (ADR), North West Mediterranean (NEW), and Aegean Sea (AEG). They correspond to the influence of large rivers, including the Nile, the Po, the Rhone and the Ebro, and the Northern Greece's rivers, respectively (MAP, 2017, Macias et al., 2018a). These hotspots are clearly detected by our model simulation of TN and TP loads, and in particular of N-NO_3_ and P-PO_4_ fluxes (Fig. 6), which represent the major anthropogenic components of nutrient export to coastal waters. In particular, the South Levantine (SLE) and the Adriatic (ADR) received about 0.83 Tg/y of N-NO_3_ and 0.016 Tg of P-PO_4_. Nitrate is the dominant component of TN in all parts of the Mediterranean Sea, while orthophosphate ranged from 7% to 39% of TP in all the Mediterranean Sea areas except for the South Levantine (67%).

### 3.3 Nutrient retention

**Fig. 7:**
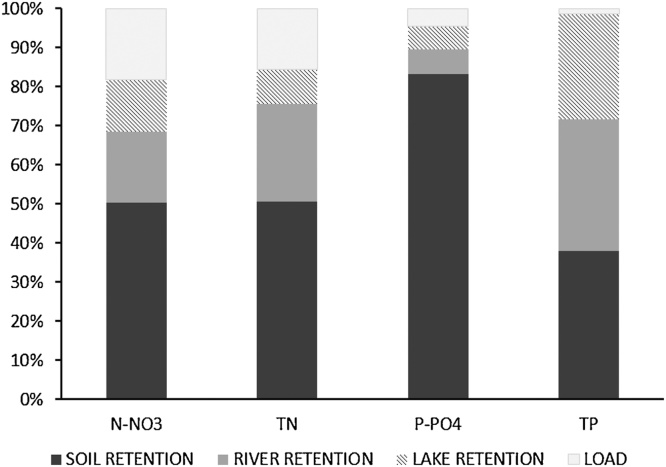
Percentages of nutrient retention and discharged loads with respect to the total inputs during the period 2003–2007.

Fig. 7 shows the percentages of nutrient retention and discharged loads into the Mediterranean Sea. These percentages were calculated comparing the sum of retained loads and discharged loads of all basins in the Mediterranean area with the total inputs.

It is noteworthy that more than 80% of total inputs are retained for TN (84%) and N-NO_3_ (81%). These percentages increase to more than 95% for P-PO_4_ and TP. The major part of retention of total nutrient inputs occurs in soils. More than 50% of diffuse sources of N-NO_3_, TN and P-PO_4_ were captured before reaching the river network, while around 38% of TP diffuse sources were retained. In rivers the retention was estimated around 18% for N-NO_3_, 25% for TN and 6% for P-PO_4_, while the reduction of TP was the highest, around 34%. Lake retention was approximately similar to that obtained for rivers, albeit slightly lower. For N-NO_3_ and TN lake retention was estimated around 13% and 9%, respectively. The lowest values were obtained for P-PO_4_ (around 6%), while the highest value was for TP (27%). These percentages are in the range of those reported in literature: for instance, Romero et al. (2016) estimates that N retained in the Mediterranean basins of the Iberian Peninsula is around 87%.

### 3.4 Source apportionment

**Fig. 8:**
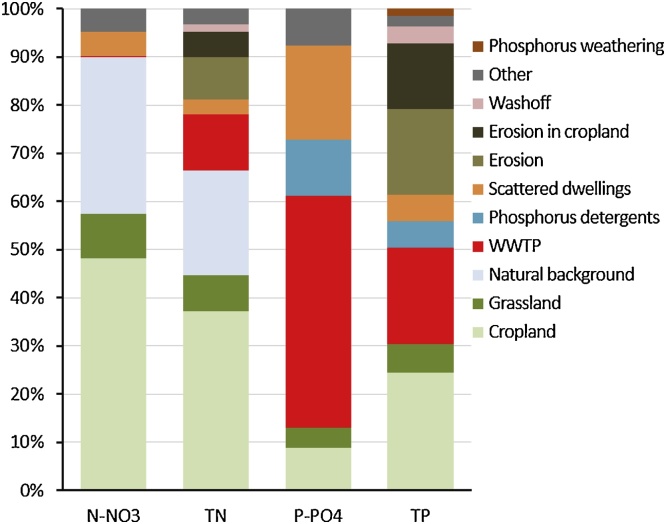
Sectoral contribution to the total discharged loads of N-NO_3_, TN, P-PO_4_ and TP for the period 2003–2007 with respect to the baseline simulation.

Nitrogen surplus in cropland and fodder constitutes the dominant source of nitrate (48%) and total nitrogen (37%) discharged into the sea, followed by natural background with 32% and 22%, respectively. Phosphate loads are mainly composed by discharge from wastewater treatment plants (48%) and by scattered dwellings (19%) (Fig. 8). For total phosphorus, agriculture and discharge from wastewater treatment plants have the highest contributions (24% and 20%, respectively), however the influence of erosion of arable soils increases the contribution of the agricultural sector to 38%.

The source apportionment for the whole Mediterranean region is mostly controlled by the situation of its main river basins including the Nile, Po, Ebro and Rhone river basins. In these areas the actions to reduce nitrogen should focus on cropland and grassland, and phosphorus needs also to be kept under control, as it might be rapidly recycled from water sediments (Garnier et al., 2010). Therefore, the control of both nitrogen and phosphorus is necessary to reduce eutrophication in coastal waters, also for the complexity of the interactions of the two elements (Howarth and Marino, 2006).

### 3.5 Potential reduction of nutrient loads in the Mediterranean

**Fig. 9:**
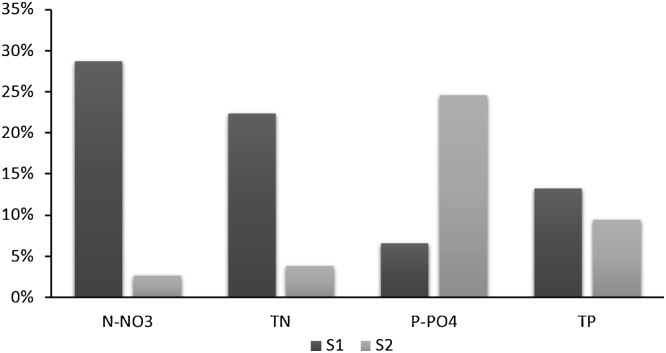
Percentages of nutrient reduction for scenarios S1 and S2 with respect to the baseline simulation of total discharged nutrient loads into the Mediterranean Sea in the period 2003–2007.

Fig. 9 shows the comparison between the nutrient reduction for scenarios S1 and S2 with respect to the baseline simulation of total discharged nutrient loads into the Mediterranean area in the period 2003–2007. Scenario S1, which simulates the reduction of nitrogen surplus on cropland land and grassland, and scenario S2, which foresees the improvement of wastewater treatments, are examples of actions to address nitrogen and phosphorus reduction in the river basins.

For the Mediterranean basins, S1 option could reduce TN by about 22%, N-NO_3_ by 29%, TP by 13% and P-PO_4_ of 7% (Fig. 9). Scenario S2 leads to a reduction of phosphates by 25%, TP by 10%, and by less than 5% for N-NO_3_ and TN.

S1 scenario is coherent with a similar scenario detailed in Thieu et al. (2012) and Bouraoui et al. (2014). In particular, our scenario S1 shows that a 50% decrease of nitrogen surplus on cropland and grassland would entail more than 20% reduction of nitrogen input to coastal waters (Fig. 9). This might appear quite a drastic scenario, but it provides a benchmark for the evaluation of impacts (for example Passy et al. (2016) simulated a scenario of 50% reduction of nitrogen input to coastal water in the Seine Bay in order to observe a drastic reduction of noxious algal blooms). In addition, even though the nitrogen surplus in European Mediterranean countries has already decreased by about 8% from 53 kg N/ha in 2005 to 48 kg N/ha in 2015 (weighted average by country area, EUROSTAT, 2018), and a strong reduction of nitrogen surplus is still desirable in some regions, such as Malta, Cyprus, and Egypt that have high nitrogen surplus (Lassaletta et al., 2014).

## 4 Discussion

In the Mediterranean Sea, intensive fishing and changes in primary productivity, driven by anthropogenic nutrient pollution, have altered the ecosystem and species biodiversity, with a reduction in abundance of important fish species and top predators compared to the 50Ys (Piroddi et al., 2017).

**Fig. 10:**
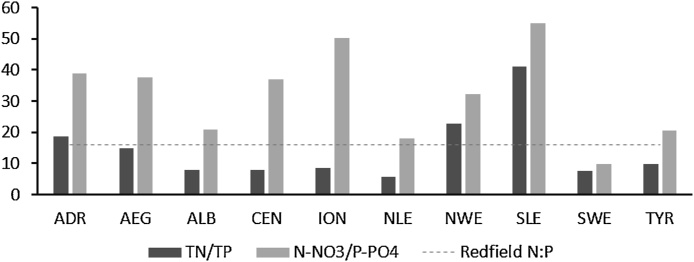
Ratio of TN/TP and N-NO_3_/P-PO_4_ in the Mediterranean Sea sub-basins.

Analyses of satellite images of the Mediterranean Sea over the last 20 years show that chlorophyll concentration (which is used as a proxy of phytoplankton biomass to detect eutrophication) has been increasing in the South-East Spanish coast, in the Ligurian–Provençal basin, and in the Rhodes Gyre region, while it has been decreasing in the North Adriatic Sea, off the Rhone River mouth, and in the Aegean Sea. The evidence also indicates that eutrophication problems persist in the area of influence of the Nile Delta (Colella et al., 2016). These trends result from the expansion of urban areas and intensification of fertilizers use in agriculture (for example in Turkey and Egypt, FAOSTAT, 2018), as well as from the implementation of policies to reduce agricultural nutrient pollution or wastewater discharges, such as in the Rhone and Po river basins, following the enforcement of the EU Nitrates Directive (91/676/EEC) and Urban Waste Water Directive (91/271/EEC). It is clear that socio-economic activities in a river basin can affect the amount and forms of nitrogen and phosphorus exported to the coastal waters, and can also alter the N:P ratio, which strongly influences the eutrophication process. According to our modelling estimates, in the period 2003–2007 the ratio TN:TP was 41, 19, 23, 15, in SLE, ADR, NWE and AEG respectively, while the ratio N-NO_3_:P-PO_4_ was 55, 39, 32, 38 (Fig. 10). The highest values of N-NO_3_:P-PO_4_ were observed in ION and SLE. As these values are higher than the Redfield N:P ratio of 16, they indicate that phosphorus can act as limiting nutrient for eutrophication, which is in line with evidence found in the literature for the Mediterranean Sea, considered generally P-limited (Tanhua et al., 2013). In addition, for the period 2001–2005 Romero et al. (2013) reported values of the ICEP index (Billen and Garnier, 2007) at the outlets of the Rhone, Ebro and Po rivers indicating a potential risk of eutrophication related to an excess of nitrogen over silica.

The persistent and widespread eutrophication processes that impact especially enclosed coastal bays in the Mediterranean Sea resulted in a huge effort in the last half century to control the phenomena. Understanding the amount of nitrogen and phosphorus discharged to the sea, their sources by a given sector, and the losses (retention) in the river basin is crucial to plan management strategies for the protection of coastal ecosystems and their services. The modelling framework presented in this study allows quantifying nutrient fluxes to the sea considering the contribution of natural processes and different sources including agriculture and domestic activities.

The estimations of soils, rivers and lakes contribution to the retention of nitrogen and phosphorus forms, like those provided in this study (Fig. 7), can help understanding the role of reservoirs, soils and river systems in the total removal of nutrients. The control of nitrogen and phosphorus losses to waters involves the understanding of the natural processes responsible for nutrient retention in the river basin. These processes depend on the mobility of the different nitrogen and phosphorus forms in water. They are mediated by the water residence time, the climate, the action of bacteria (denitrification), the plants and algae growth, and the processes of sedimentation (especially in standing waters and in lakes) (Grizzetti et al., 2015). Maintaining and enhancing natural riparian filters supports nutrient retention processes in the river network (Viaroli et al., 2015, Soana et al., 2017), while intensification of dams in the Mediterranean region can increase retention but at the same time expand nitrogen pollution in aquifers and soils (Romero et al., 2016).

In reference to the major challenges that the Mediterranean area will face in future years, i.e. intensification of economic activities, population increase, urban sprawling and climate changes, the scenarios S1 and S2 of this study provided significant management options. In particular, they provide the potential effectiveness of mitigation measures on the various forms of nitrogen and phosphorus. It is noteworthy that due to the structure of GREEN-Rgrid we can assess trade-offs scenarios in particular those linked to change of land use and change of irrigated areas and associated impacts on crop production and fertilizer requirements.

Despite the current conflict in Syria and the related political instability of some countries of the area, economic activities and population are expected to increase in North Africa and Middle East. The expansion of the economic sectors and agriculture may increase water demand by 18% by 2025, mainly in the Southern and Eastern countries (Plan Bleu, 2008). The coastal urban population in the Mediterranean is projected to increase by 30% from 2005 to 2025, the increase will take place mainly in urban areas in the Eastern and Southern coasts of the Mediterranean (Plan Bleu, 2008). All these aspects could exacerbate the current pressure on the rich and fragile ecosystem that sustains the people and economic activities of the region (EEA, 2015). Concurrently, in the Mediterranean area the climate is expected to be warmer and drier by the end of the century (IPCC, 2013), this will reduce water availability and affect nutrient cycling. In coastal regions the increase of temperature and the change in precipitation patterns could foster the growth of phytoplankton biomass (Macias et al., 2018b).

As a consequence of the population increase, higher direct discharges of nutrient through sewage systems can be expected in the Mediterranean coasts. Under a growing population, upgrading wastewater treatment to tertiary level, especially for direct discharges into the sea, is a necessary measure to protect the marine and coastal environment. Powley et al. (2016) estimated nitrogen and phosphorus wastewater directly discharged by coastal cities into the Mediterranean Sea. Their scenario indicates that treating these discharges to tertiary level would be needed to keep pollution in 2050 at the same level of 2003, despite the population increase, but it would involve additional costs over 2 billion €/y. Our scenario S2, which considers the contribution of wastewater from coastal cities and also from the whole drainage basin, shows that a 10% reduction of TP input could be achieved by upgrading the existing wastewater treatment facilities (Fig. 9). This reduction could be up to 30% in the coastal areas of Northern African and Asian basins, such as in the Nile and Turkish basins, where chlorophyll increasing concentrations have been observed (Colella et al., 2016).

Reducing nitrogen surplus on agricultural soils together with collecting wastewaters and upgrading treatment levels, as exemplified by scenarios S1 and S2 in this study, are necessary measures to protect the water resources and coastal and marine ecosystems in the Mediterranean basin, and to ensure the long-term sustainability of the economic activities and ecosystem services in the region. These measures are also relevant in view of the future climatic and demographic challenges the region will face regarding nutrients in the agriculture-water nexus.

In conclusion, the assessment and modelling tool presented in this study have shown how the interlinkage between water and agriculture can be explored, considering the basin-coastal relationship and nutrient sources and sinks from different sectors in an integrated way, making it a valuable tool not only for the Mediterranean area but also for all the other seas.
