# Supplementary material for: Modelling nutrient fluxes into the Mediterranean Sea
Source: J Hydrol Reg Stud. 2019 Apr;22:100592. doi: 10.1016/j.ejrh.2019.01.004 (PMC6472532; doi:10.1016/j.ejrh.2019.01.004)
Supplement: Supplementary file 1 [file mmc1.docx]

*Journal of Hydrology: Regional Studies*

Supporting Information for

Modelling nutrient fluxes into the Mediterranean Sea

Anna Malagò^1^, Faycal Bouraoui^1^, Bruna Grizzetti^1^ and Ad De Roo^1^

1 European Commission, Joint Research Centre (JRC), Ispra, Italy

Corresponding author: Anna Malagò ([anna.malago@ec.europa.eu)](mailto:anna.malago@ec.europa.eu))

**Contents of this file**

Tables S1

**Introduction**

The GLOBCOVER 2009 map (Arino et al., 2008) with spatial grid resolution of about 200-300 m was used to define 10 classes of landcover: crop land (ARAB), fodder (FODG), grass land (GRAS), forest (FRST), shrub (SHRU), bare (BARE), urban area (URHD), water (WATR), sea (WSEA) and snow (SNOW). These classes were summarized in grid cells of 5 minutes at global scale. The extent of the class crop land was fixed using the information of used agricultural land reported in the Spatial Production Allocation Model (SPAM) at 5 minutes resolution. The class fodder was then obtained as the difference between the aggregated classes chosen as representative of the arable land and the agricultural land of SPAM.

The basic data to characterize pressure coming from agriculture is the output of the Spatial Production Allocation Model (SPAM; You et al., 2014). The model was developed by IFPRI to generate crop areas, crop yield at 5 arc-minute resolution using all relevant spatial explicit background information including “national and sub-national crop production statistics, satellite data on land cover, maps of irrigated areas, biophysical crop suitability assessments, population density, secondary data on irrigation and rain fed production systems, cropping intensity, and crop prices” (You et al., 2014). More specifically it provides for 42 crops and four levels of intensification the physical area where a crop is grown, the harvest area for a specific crop to consider multiple-harvest in a specific year, the yield and the production (product of yield and harvest area). Data are provided for year 2005 (average of 3 years centred on 2005) for four production systems including irrigated high inputs production, rainfed high inputs production, rainfed low inputs production, rainfed subsistence production (You et al., 2014).

In the GREEN-Rgrid, the nitrogen and phosphorous uptake from crops and fodder grazing was obtained by multiplying the crop dry yield from SPAM by the corresponding crop coefficients listed in Table S1. Instead, the uptake from grassland areas was calculated as 60% of nutrients inputs (Bouwman et al., 2005).

The soil organism fixation for each crop used in this study is reported in Table S1. For fodder the fixation was set to 65 kg/ha, while for grassland to 4 kg/ha.

In particular, the nitrogen fixation for each crop was calculated based on the harvest area of each crop.

Table S1. SPAM crops and coefficients used in this study, where CNYLD and CPYLD are respectively the nutrient content of the crop (kg N or P/kg yield); FIX is the specific nitrogen fixation (kg/N/ha) and MC is the moisture content in percentage.

| Code | Description | CNYLD* | CPYLD* | FIX**  (kg/ha) | MC (%)*** |
| --- | --- | --- | --- | --- | --- |
| acof | arabica coffee | 0.0015 | 0.0003 | 4 | 60 |
| bana | banana | 0.0064 | 0.0008 | 4 | 74 |
| barl | barley | 0.021 | 0.0017 | 4 | 12 |
| bean | bean | 0.037 | 0.0021 | 40 | 12 |
| cass | cassava | 0.0097 | 0.001 | 4 | 80 |
| chic | chickpea | 0.0427 | 0.0048 | 60 | 12 |
| cnut | coconut | 0.0015 | 0.0003 | 4 | 45 |
| coco | cocoa | 0.0015 | 0.0003 | 4 | 60 |
| cott | cotton | 0.014 | 0.002 | 4 | 1 |
| cowp | cowpea | 0.0427 | 0.0048 | 60 | 12 |
| grou | groundnut | 0.0505 | 0.004 | 80 | 6 |
| lent | lentil | 0.0506 | 0.0051 | 60 | 12 |
| maiz | maize | 0.014 | 0.0016 | 4 | 15 |
| ocer | other cereals | 0.0316 | 0.0057 | 4 | 10 |
| ofib | other fibre crops | 0.04 | 0.0033 | 4 | 12 |
| oilp | oilpalm | 0.0019 | 0.0004 | 4 | 30 |
| ooil | other oil crops | 0.0015 | 0.0003 | 4 | 60 |
| opul | other pulses | 0.037 | 0.0021 | 60 | 12 |
| orts | other roots | 0.0097 | 0.001 | 4 | 80 |
| pige | pigeonpea | 0.0427 | 0.0048 | 60 | 12 |
| plnt | plantain | 0.0064 | 0.0008 | 4 | 70 |
| pmil | pearl millet | 0.02 | 0.0028 | 4 | 12 |
| pota | potato | 0.0246 | 0.0023 | 4 | 80 |
| rape | rapeseed | 0.0234 | 0.0033 | 4 | 8.5 |
| rcof | robusta coffe | 0.0015 | 0.0003 | 4 | 60 |
| rest | rest of crops | 0.0199 | 0.0032 | 4 | 10 |
| rice | rice | 0.0136 | 0.0013 | 25 | 14 |
| sesa | sesameseed | 0.0019 | 0.0004 | 4 | 30 |
| smil | small millet | 0.02 | 0.0028 | 4 | 12 |
| sorg | sorghum | 0.0199 | 0.0032 | 4 | 10 |
| soyb | soybean | 0.065 | 0.0091 | 80 | 13 |
| sugb | sugarbeet | 0.013 | 0.002 | 4 | 80 |
| sugc | sugarcane | 0.0069 | 0.0017 | 4 | 77 |
| sunf | sunflower | 0.0454 | 0.0074 | 4 | 6 |
| swpo | sweet potato | 0.0097 | 0.001 | 4 | 80 |
| teas | tea | 0.0015 | 0.0003 | 4 | 75 |
| temf | temperate fruit | 0.0019 | 0.0004 | 4 | 84 |
| toba | tobacco | 0.014 | 0.0016 | 4 | 10 |
| trof | tropical fruit | 0.0019 | 0.0004 | 4 | 87 |
| vege | vegetables | 0.0259 | 0.0031 | 4 | 93 |
| whea | wheat | 0.0234 | 0.0033 | 4 | 12 |
| yams | yams | 0.0097 | 0.001 | 4 | 80 |
| FRST | forest | 0.0015 | 0.0003 | 4 | 0 |
| FODG | fodder grazing | 0.01 | 0.002 | 65 | 73 |

* Neitsch et al. (2010)

** Liu et al. (2010); Grizzetti et al. (2006)

***Williams (1995)

References

Arino, O., Bicheron, P., Achard, F., Latham, J., Witt, R., & Weber, J. L., 2008. Globcover: the most detailed portrait of Earth, ESA Bulletin 136, 24-31.

Bouwman, A. F., G. Van Drecht, & K.W.Van der Hoek, 2005. Nitrogen surface balances in intensive agricultural production systems in different world regions for the period 1970 2030. Pedosphere 15,137–155.

Grizzetti, B. and Bouraoui, F., 2006. Assessment of Nitrogen and Phosphorus Environmental Pressure at European Scale. Report EUR 22526 EN. pp.66.

Liu J., You L., Amini M., Obersteiner M., Herrero M., Zehnder A. J .B. and Yang H., 2010. A high-resolution assessment on global nitrogen flows in cropland. Proc. Natl. Acad. Sci. USA, 107 8035–40.

Neitsch, S. L., Arnold, J. G., Kiniry, J. R., Srinivasan, R., and Williams, J. R., 2010. Soil and Water Assessment Tool Input/Output File Documentation Version 2009, Grassland, Soil and Water Research Laboratory, Agricultural Research Service and Blackland Research Center, Texas Agricultural Experiment Station, College Station, Texas, 2010.

Williams, J.R., 1995. The EPIC model. In: Computer models of watershed hydrology editors Singh, V.P. Water Resources Publications, Highlands Ranch, CO, USA, 909-1000.

You, L., Wood,S., Wood-Sichra, U., Wu, W., 2014. Generating global crop distribution maps: From census to grid. Agricultural Systems 127, 53-60.
